# Detection of the Spatio-Temporal Differentiation Patterns and Influencing Factors of Wheat Production in Huang-Huai-Hai Region

**DOI:** 10.3390/foods11111617

**Published:** 2022-05-30

**Authors:** Yifan Zhang, Bingjun Li

**Affiliations:** College of Information and Management Science, Henan Agricultural University, Zhengzhou 450046, China; zyf185423@163.com

**Keywords:** wheat production, spatio-temporal differentiation, SCA-GWR, influencing factor, Huang-Huai-Hai

## Abstract

The stability of wheat production is closely related to national food security and agricultural sustainable development, and it has been a major policy concern for China. By analyzing the spatiotemporal factors and causes of wheat production, we can grasp the spatiotemporal distribution law of wheat production to rationally allocate agricultural resources. To this end, this study first conducted a quantitative analysis of the yield differentiation patterns in Huang-Huai-Hai (HHH) wheat based on the 2010–2020 wheat agricultural data, comprehensively using the Theil index and exploratory spatial data analysis. Second, to eliminate the spatial heterogeneity and multicollinearity of the modeling variables, a local model of SCA-GWR combining Spearman correlation analysis (SCA) and geographically weighted regression (GWR) was established. Compared with the traditional global regression model, the superiority and applicability of the SCA-GWR model are proved, and it is a simple and effective new method to detect spatial data nonstationarity. Finally, the factors influencing wheat production in the HHH region were detected based on the SCA-GWR local model, and relevant policy recommendations were put forward. The results show that: (1) The yield difference in different farming areas gradually narrowed, and the wheat production had a significant High-High aggregation trend. The center of gravity for wheat production lies in the southwest of the HHH region. (2) Wheat production still has a strong dependence on irrigation and fertilizer. Effective irrigated areas and temperature are the main driving forces for its production. The inhibitory effect of the proportion of nonagricultural employment on wheat production gradually weakened. Radiation and rainfall were only significantly positively correlated with wheat production in the central and southern HHH region. In response to the findings of the study, corresponding policy recommendations are made in terms of optimizing the allocation of resources, increasing investment in agricultural infrastructure, and vigorously researching and developing agricultural science and technology, and the results of the study can provide a basis for decision-making and management by relevant departments.

## 1. Introduction

Wheat (*Triticum aestivum* L.) is one of the oldest cultivated crops in the world and is the staple food for about 40% of the world’s population [1]. From 2010 to 2020, the yield of HHH wheat accounted for more than 60% of the total national grain inventory for eleven consecutive years [2,3]. As the largest wheat-producing area in China, the HHH region has an increasingly prominent status as a food hub and plays a pivotal role in ensuring national food security. However, in recent years, the excessive input and unreasonable distribution of agricultural production factors such as pesticides and fertilizers in the region have severely restricted food security and have become a bottleneck for sustainable agricultural development [4]. It can be seen that clarifying the spatial pattern of wheat production in the HHH, detecting the major and minor factors affecting its yield, and reasonably allocating the factors of agricultural production are of great significance for the formulation of a reasonable scientific wheat production policy and sustainable agricultural development.

Regarding the research on the spatiotemporal differentiation patterns of wheat production, Gini coefficient, industrial concentration index, Theil index, etc. are widely used [5,6,7]. Wu et al. [8] believed that China’s wheat production fluctuate once every 7 years on average. Feng et al. [9] and Li et al. [10] quantified the spatiotemporal pattern of wheat production from the perspective of wheat yield increase and the evolution of wheat production efficiency, respectively. Guo et al. [11] analyzed the spatial pattern of wheat yield using cluster analysis and found that while wheat production is improving in the time dimension, it is also necessary to pay attention to the gap in the space dimension. In the detection of factors affecting wheat production, research techniques mainly include the spatial Durbin model [12], stepwise regression analysis [13], farmer field surveys [14], and machine learning [15]. A more consistent conclusion is obtained: the impact of a crop yield is multifaceted and three-dimensional [16]; Climatic factors are the most direct factors affecting wheat yield and causing its regional differences [17,18]. Most of the results obtained with the crop simulation model show that chemical fertilizer application and irrigation level have positive effects on wheat production, while mechanical input and technical level have negative effects [19]. Wang and Xiao [20] used a model of seemingly irrelevant equations and found that factor input and social economy are the driving factors affecting the spatial pattern of wheat production. Hao et al. [21] and Li [22] both used the spatial Durbin model and found that the optimization of wheat production layout is a fundamental guarantee for consolidating the foundation of sustainable agricultural development. Zhang et al. [23] revealed the main factors for the increase in winter wheat yield in different regions of the Loess Plateau. It can be seen that the formation of the spatial pattern of wheat is the result of the comprehensive effect of various conditions.

The research results of scholars are significant, and they serve as a good reference for the development of this research. However, domestic and foreign scholars have not considered the spatial dependence of wheat production layout, and it is rare to analyze the production patterns and driving factors together. Related studies have stopped at the quantitative analysis of the whole field. Due to differences in geographical environment and natural conditions, the same factor often produces different effects in different regions. How to overcome the non-equilibrium of geospatial data and analyze the regional limiting factors affecting the yield of winter wheat, the existing research has rarely reported on this aspect.

In addition, the inequitable allocation of social resources caused by the imbalance of natural resource endowments and socioeconomic conditions often leads to spatial heterogeneity [24]. The spatial correlation of factors such as economic development and population mobility often leads to strong correlations between cross-regional wheat production. However, the traditional ordinary least squares (OLS) regression model ignores the spatial factors of wheat production, so its results cannot explain the spatial evolution of wheat production [25]. Furthermore, although local regression models such as spatial error and spatial lag models consider spatial factors, they often only consider the correlation of spatial factors and fail to examine the spatial variation in the influencing factors in different regions from the perspective of spatial heterogeneity [26]. These facts fully explain the necessity of detecting the influencing factors of the HHH wheat based on spatial heterogeneity.

Geographical weighted regression (GWR) was proposed by British scholar Fotheringham in 1996 as an effective modeling technique for addressing the phenomenon of nonstationarity of data in regression analysis, and it is an important spatial statistical method to quantify spatial heterogeneity [27]. This method introduces the varying spatial position coordinates into the regression model, and the estimated value of the variable coefficient changes with changes in spatial position. Compared with OLS regression, the GWR model fully considers spatial heterogeneity and spatial dependence. In fact, this model has been widely studied in ecological land use [28], disaster management [29], and food security [30], but it is rarely used to detect the influencing factors of wheat production. To avoid multicollinearity in the model and instability of the analytical results, and to improve the simplicity of the model, Spearman correlation analysis and GWR were combined to construct an SCA-GWR local model based on the GWR model. This model effectively improves the generalization ability of the GWR model, taking into account the stability of regression parameters and the nonstationarity of spatial data. The validity of the model will be further verified by comparative analysis with global models such as traditional OLS.

In view of this, this study focuses on exploring the issue of spatial differentiation pattern in wheat production in conjunction with the Thiel index and exploratory spatial data analysis. The SCA-GWR local model was developed to reveal the main factors limiting wheat yield improvement in different regions of the HHH under present-day management conditions and the spatial and temporal variability of their influences. The study proposes the following hypotheses: There is multicollinearity among the explanatory variables in the regression model; there is a certain spatial heterogeneity in the influencing factors of wheat production. The aim of the study is to provide a theoretical basis for the scientific management of winter wheat production in the HHH, to provide basic information for food security and sustainable regional agricultural development, and to provide new methods to explain the spatial and temporal mechanisms of factor action.

## 2. Materials and Methods

### 2.1. Overview of the Study Area

**Figure 1:**
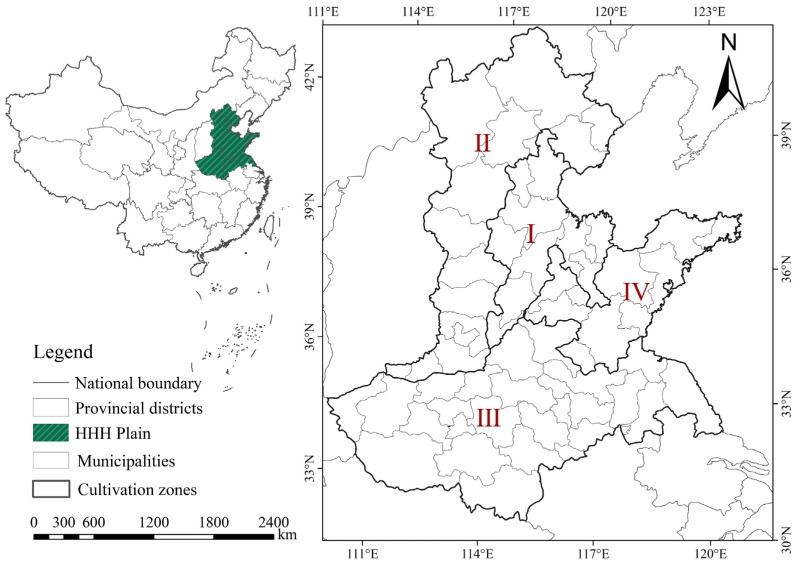
Study location overview map. I: Hebei, Shandong, and Henan low-lying plains; II: pre-mountain plain area in Yanshan and Taihang Mountains; III: Huang-Huai plain area; IV: Shandong hilly area.

The HHH plain is an important grain-producing area in China, being the “golden area” for winter wheat in China (Figure 1) [31]. The HHH agricultural area is located in eastern central China, with an area of about $3.2\times 10^{3}$ km^2^. This area belongs to the warm temperate semi-humid climate, with sufficient sunlight and fertile land [32]. Its unique geographic location, climate, and soil conditions provide the best environment for wheat growth. The perennial wheat sowing area is about $1.3\times 10^{7}$ hm^2^, accounting for about 56% of the national winter wheat area, and the total yield is about $7.375\times 10^{8}$ tons, accounting for more than 67% of the national total [33]. It can be seen that the stability of wheat production in the HHH plain is directly related to national food security and sustainable social development.

### 2.2. Data Sources and Processing

Wheat yield is the result of the combined action of natural factors and socioeconomic factors. The data types chosen therefore include climatic meteorological data (natural attributes), socioeconomic data (social attributes), and some map vector data (spatial attributes). The data range is 2010–2020. Among them, the geographic data were 1:800,000 HHH municipal administrative divisions. Taking full consideration of the availability of data, the selection of natural attribute and social attribute data is as follows.

#### 2.2.1. Natural Attribute Data

**Figure 2:**
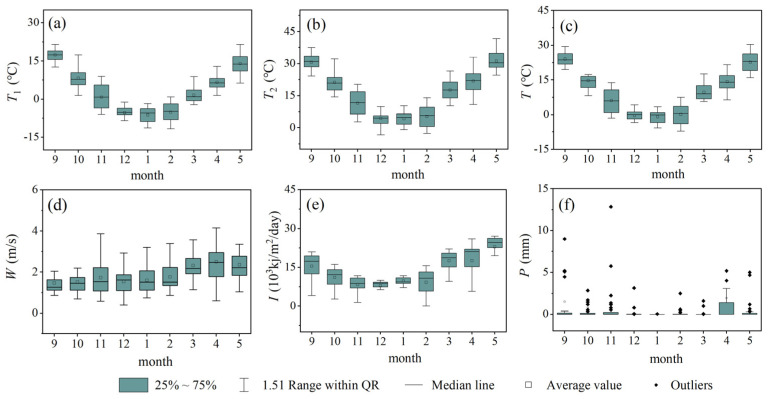
Distribution of meteorological variables. (**a**) *T_1_*: Daily minimum temperature. (**b**) *T_2_*: Daily maximum temperature. (**c**) *T*: Daily average temperature. (**d**) *W*: Average wind speed. (**e**) *I*: Solar radiation. (**f**) *P*: Average precipitation.

The meteorological data came from the China Meteorological Data Network (http://data.cma.cn/, accessed on 1 December 2021), from which 15 agricultural meteorological observation stations with continuous meteorological observation data, typical representatives, and uniform spatial distribution were selected. Due to the huge amount of meteorological data, we use Origin software to calculate the descriptive statistics for each variable by month (Figure 2) and through the inverse distance weight method for spatial interpolation. Among them, *T_1_*, *T_2_*, *T*, *W*, and *I* have obvious distribution characteristics in different growth periods of wheat. Since *P* is mostly concentrated in a specific period of the year, and there is no specific law in the same period, the distribution of *P* is mostly points of the discrete state.

#### 2.2.2. Social Attribute Data

Total wheat yield was used to characterize wheat production. In addition to the factors that cannot be determined, according to the basic principles of the feasibility, comprehensiveness, and quantitative and qualitative combination of index selection, 8 social factors including weighted agricultural machinery total power are selected as economic condition factors. The weighted variable weight is ω (current wheat sown area/current crop sown area). Data of relevant indicators were obtained from the statistical yearbooks of the HHH provinces and the National Bureau of Statistics (http://www.stats.gov.cn/, accessed on 1 December 2021) from 2011 to 2021. Table 1 presents statistical information on the variables represented by the years 2010 and 2020, in the same units as the corresponding variables, and the statistical results are reported and accounted for the study data.

### 2.3. Research Methods

#### 2.3.1. Theil Index

The Theil index is often used to measure regional differences in the context of multiple research units. Its advantage lies in that it can effectively reflect the spatial differences of wheat yield within and between regions and clarify its contribution to the overall difference [34]. The formula is as follows:

(1) $$T_{theil}=\sum \frac{y_{m}}{Y}\times \mathrm{lg}\left((y_{m}/Y)/\left(x_{m}/X\right)\right)$$

(2) $$T_{inter}=\sum \frac{y_{j}}{Y}\times \mathrm{lg}\left(\left(y_{j}/Y\right)/\left(x_{j}/X\right)\right)$$

(3) $$T_{i}=\sum \frac{y_{m}}{Y}\times \mathrm{lg}\left(\left(y_{m}/Y_{j}\right)/\left(x_{m}/X_{j}\right)\right)$$

where $T_{theil}\in \left[0,1\right]$ is the Theil index; $x_{m}$ and $y_{m}$ respectively represent the yield level of m city in the i wheat planting division; X and Y are the sown area and yield of wheat in the HHH region, respectively; $X_{j}=\sum x_{m}$ and $Y_{j}=\sum y_{m}$ are the sown area and yield of the i wheat planting division, respectively; $T_{inter}$ represents the interval difference; and $T_{i}$ represents the intra-regional difference of the i wheat planting division.

#### 2.3.2. ESDA

Exploratory spatial data analysis (ESDA) can effectively diagnose the spatial distribution patterns of wheat yield [35]. ESDA is used here to explore the spatial differences between different cultivation zones in the HHH. The spatial correlation of regional wheat yield is reflected by Moran’s I. The formula is expressed as follows:

(4) $$I=n\sum_{i=1}^{n}\sum_{j\neq i}^{n}W_{ij}\eta_{i}\eta_{j}/\left(\left(\sum_{i=1}^{n}\sum_{j=1}^{n}\omega_{ij}\right)\sum_{i=1}^{n}\eta_{i}^{2}\right)$$

(5) $$I_{i}=\eta_{i}\sum_{j\neq i}^{n}\omega_{ij}\eta_{j}/\left(\sum_{i=1}^{n}\eta_{i}^{2}/n\right)\;$$

In Equation (4), n is the number of city areas; $\eta_{i}=d_{i}-\bar{d}$,$\eta_{j}=d_{j}-\bar{d}$, $d_{i}$ and $d_{j}$ represent the wheat yield of the i city and the j city unit, respectively; $\bar{d}$ is the average yield of all cities; $\omega_{ij}$ is the spatial weight value, and $\omega_{ij}$ is determined by the spatial weight matrix W. In Equation (5): $I_{i}$ represents the local Moran’s I of i region, which quantifies the spatial aggregation relationship of the city area unit and can cluster the spatial data into four aggregation forms: High-High, High-Low, Low-Low, and Low-High.

#### 2.3.3. SCA-GWR

Regression analysis is the most basic and important tool in data analysis. Identifying important variables, judging the degree and direction of correlation, and using regression coefficients to estimate weights are the three important missions of regression analysis [36]. Here, the size and direction of the regression coefficients of the geographically weighted regression (GWR) model are mainly used to diagnose the influencing factors. Compared with the traditional OLS model, the GWR model considers spatial factors, and its results can better reflect local characteristics [37]. In addition, the test results show that regression models involving all the above variable combinations will lead to severe collinearity problems, and the estimated results may lose their explanatory significance [38]. Due to the strong correlations between socioeconomic factors, improper selection of variables will lead to multicollinearity, which not only increases the complexity of the model but also leads to unstable analysis results. Therefore, the Spearman correlation analysis and the GWR model are comprehensively applied here, and the SCA-GWR local model is constructed and applied to the diagnosis of wheat production problems in HHH. The modeling steps of the SCA-GWR model are as follows:

- *Step 1*. Screen variables from the SCA.

Spearman’s correlation coefficient was used to quantify the degree and direction of the linear relationships between variables. The formula for calculating the Spearman correlation coefficient is:

(6) $$r_{s}=1-6\sum_{i=1}^{n}d_{i}^{2}/n\;(n^{2}-1)$$

Among the variables, $r_{s}\in \left(-1,1\right)$, $d_{i}$ is the grade difference between $X_{i}$, and the rank of a number is defined as the position of the number after the variable sequence where the number is located is sorted from small to large.

- *Step 2*. Establish the GWR model.

The GWR model incorporates spatial factors, and the standard errors of the model coefficients measure the reliability of the estimates for each coefficient. The model structure is:

(7) $$y_{i}=\beta_{0}\left(u_{i},v_{i}\right)+\sum_{k=1}^{p}\beta_{k}\left(u_{i},v_{i}\right)x_{ik}+\varepsilon_{i}$$

In the equation, $y_{i}$ refers to the fitting value of region i; k is the number of independent; variables; $x_{ik}$ is the value of the k independent variable in area i; $\left(u_{i},v_{i}\right)$ is the geographic center coordinates of area i; $\beta_{k}\left(u_{i},v_{i}\right)$ is the value of continuous function $\beta_{k}\left(u,v\right)$ in region i; and $\varepsilon_{i}$ is the random error term for region i.

- *Step 3*. Estimate the parameters.

The parameter estimates for sample i are given by the decay function:

(8) $$\hat{\beta }\left(u_{i},v_{i}\right)={\left(X^{T}W\left(u_{i},v_{i}\right)X\right)}^{-1}X^{T}W\left(u_{i},v_{i}\right)Y$$

In the equation, $W\left(u_{i},v_{i}\right)$ is the spatial weight matrix [39], which is the conceptualization of the spatial relationship. The spatial weight function generally adopts the double square function, which is a combination of the distance threshold method and the Gaussian function. The formula is:

(9) $$W_{ij}=\{\begin{array}{cc} {\left(1-{\left(d_{ij}/b\right)}^{2}\right)}^{2}, & d_{ij}\leq b\\ 0 & ,d_{ij}>b \end{array}$$

The choice of bandwidth is particularly important. Cross-validation (CV) is used to determine the size of the bandwidth, as shown in Equation (10), when the minimum corresponds to the best bandwidth.

(10) $$CV=\sum_{i=1}^{n}{\left[y_{i}-y_{\neq i}\left(b\right)\right]}^{2}$$

- *Step 4*. Inspect accuracy.

The accuracy of the model was evaluated using the determination coefficient $R^{2}$ and the Akaike information criterion (*AIC*) at the same time. $R^{2}$ is expressed as:

(11) $$R^{2}=1-\frac{\sum_{i=1}^{n}{\left(y_{j}-d_{j}\right)}^{2}}{\sum_{i=1}^{n}{\left(y_{j}-\bar{y_{j}}\right)}^{2}}$$

*AIC* is a standard to measure the goodness of model fitting, and it can also estimate the complexity of the model, taking into account the simplicity and accuracy when evaluating the model [40]:

(12) AIC=2k+nln(RSS/n)

where n is the sample size, RSS is the residual sum of squares, and k is the number of variables in the model.

The SCA-GWR model quantifies spatial heterogeneity and is an extension of tradition OLS. It not only eliminates the hidden danger of multicollinearity but also embeds the geographic location coordinates $\left(u_{i},v_{i}\right)$ of the sample point data into the regression parameters, so that each sample space unit corresponds to a coefficient value and the model results can better reflect the local characteristics. Based on variable screening, SCA-OLS, SCA-SEM, and SCA-SLM models can be established in the same way, and the superiority of the SCA-GWR model can be verified by establishing the HHH wheat production factor model.

## 3. Results

### 3.1. Temporal and Spatial Differentiation of Wheat Production in the HHH

#### 3.1.1. Time Distribution

**Figure 3:**
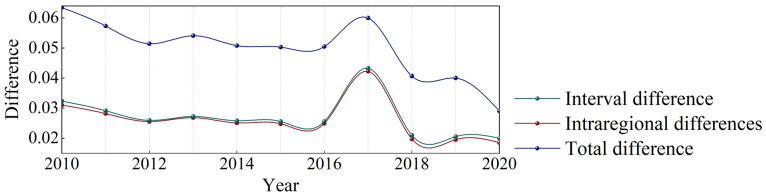
Evolution and decomposition of wheat yield differences in the HHH region.

**Figure 4:**
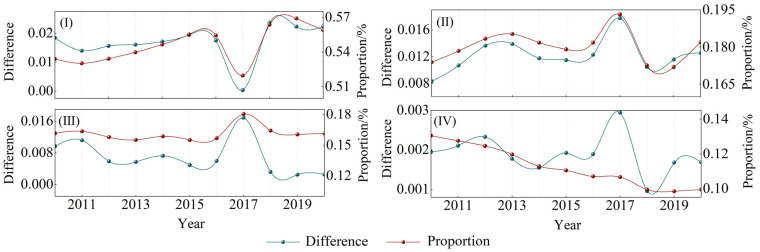
Evolution and decomposition of wheat yield differences in different cultivation areas in the HHH region. (**I**) Hebei, Shandong, and Henan low-lying plains. (**II**) pre-mountain plain area in Yanshan and Taihang Mountains. (**III**) Huang-Huai plain area. (**IV**) Shandong hilly area.

According to the Theil index Equations (1)–(3), the overall difference in the HHH wheat production from 2010 to 2020 gradually narrowed, and the regional differences kept pace and gradually narrowed, and the difference increased sharply in 2017 (Figure 3). The overall difference of the HHH wheat is composed of the differences between the four secondary farming areas and the differences within the secondary area (Figure 4).

Figure 4 shows that the differences in wheat production in zones II, III and IV are consistent, and they all experienced a process of gradually narrowing. Only the wheat difference in zone I showed a V-shaped trend with 2017 as the lowest point. Contributions to the overall variance vary by district: From a lateral perspective, zone I contributed the most to the overall difference in the HHH, with an average contribution of 54.47%, followed by zones IV and III, and zone IV had the lowest contribution rate (below 15%). From a longitudinal perspective, the internal differences between zones I and II gradually increased, which together led to an increase in the contribution of regional differences to the overall differences from 70.79% to 74.10%. The internal differences between zones III and IV gradually decreased, which together led to an increase in the contribution of regional differences to the overall differences from 29.21% to 25.90%.

#### 3.1.2. Spatial Distribution

**Figure 5:**
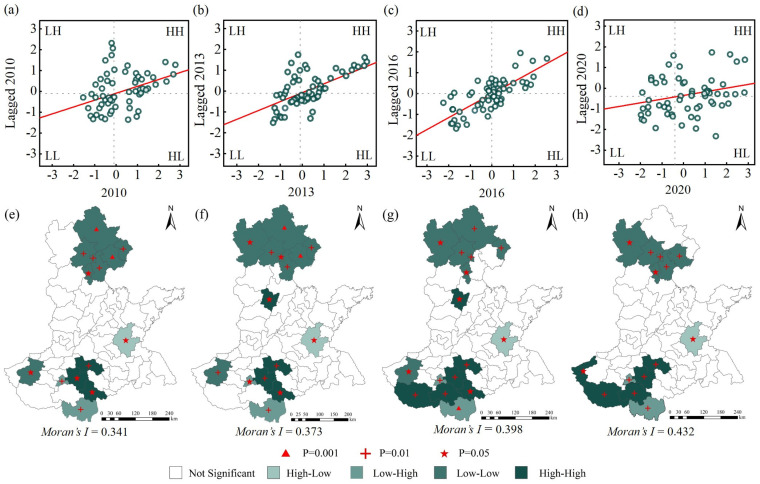
Moran scatter plot and LISA aggregation plot for wheat production in the HHH: (**a**–**d**) correspond to Moran scatter plots of wheat production in 2010, 2013, 2016, and 2020, respectively; (**e**–**h**) correspond to LISA aggregation maps for wheat production in 2010, 2013, 2016, and 2020, respectively. HH, LL, HL, and LH indicate that wheat yields show a clustering pattern of High-Low, Low-Low, High-Low and Low-High, respectively.

According to Equations (4)–(5), the spatial agglomeration of wheat yield was visually analyzed. In Figure 5, Moran’s I > 0, all years pass the significance test, and the two changes largely coincide with each other compared to Figure 4. The Moran scatter plot in Figure 5 is an initial determination of the quadrant to which the sample points belong, while the LISA aggregation plot enables an overall determination of the type of local correlation in each region and whether its areas of aggregation are statistically significant. Analysis of the four aggregation types High-High (HH), Low-Low (LL), High-Low (HL) and Low–High (LH) of the spatial pattern of wheat yields revealed that: HH types are mainly concentrated in zone I, radiating out to surrounding counties and cities from Zhumadian, which is a “hot spot” for grain production. The LL type is concentrated in the areas of Hengshui and Cangzhou in zone III. With the improvement of agricultural inputs and the vigorous transformation of saline-alkali land, it is believed that the situation of low wheat yield will be gradually eliminated in the future. HL and LH are the least distributed and not concentrated. Among them, the wheat yields in Linyi and Xinyang have been clustered in HL and LH, respectively, in the study year. The wheat yields in these regions are quite different from their surrounding areas, and the reason is that the economic conditions are uneven.

**Figure 6:**
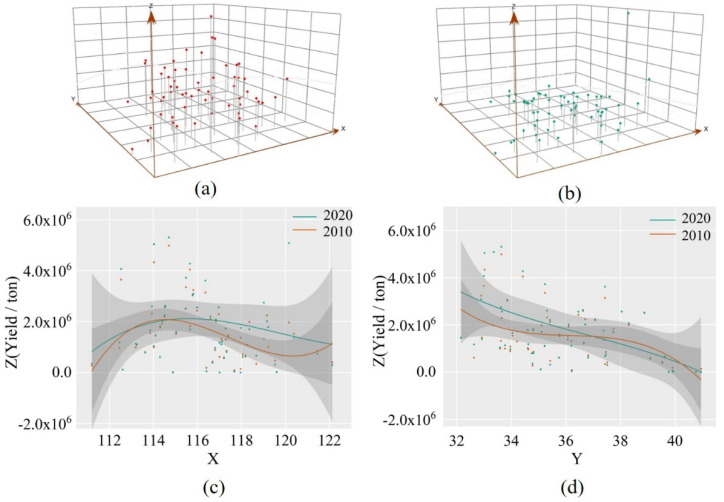
Spatial trend analysis of wheat production in the HHH City. (**a**) The spatial trend of wheat production in 2010. (**b**) the spatial trend of wheat production in 2020. (**c**) trends in wheat yield along the *x*-axis. (**d**) trends in wheat yield along the *y*-axis.

Using ArcGIS’s Geostatistical Analyst ArcToolbox, the spatial distribution trend of wheat yield in the HHH cities in 2010 and 2020 was obtained as shown in Figure 6. Each vertical line in Figure 6a,b represents the wheat yield information of a city. The *X*-axis and *Y*-axis represent the east-west and north-south geographic directions respectively, that is, the longitude and latitude of spatial geographic coordinates, and the *Z*-axis represents the wheat yield. The green and orange curves in Figure 6c,d represent the fitting trend of wheat yield in the *X*-axis and *Y*-axis directions in the HHH cities. The fitted curve shows that the HHH wheat yield has not changed significantly in the past two years, but the average yield in the eastern part of the HHH is slightly higher than that in the west, and the average wheat yield in the western part is gradually higher than that in the eastern part, which was consistent with the research result that the yield center of the HHH wheat was in Henan Province [41]. It can be seen that the current wheat production areas are more often located in less economically developed areas, these areas have a certain gap between agricultural inputs, infrastructure construction, farmers’ education level, and economic development; wheat production is weak, which is not conducive to the sustainable development of the wheat industry.

### 3.2. HHH Wheat Production Factor Model Establishment

#### 3.2.1. Variable Filtering

**Figure 7:**
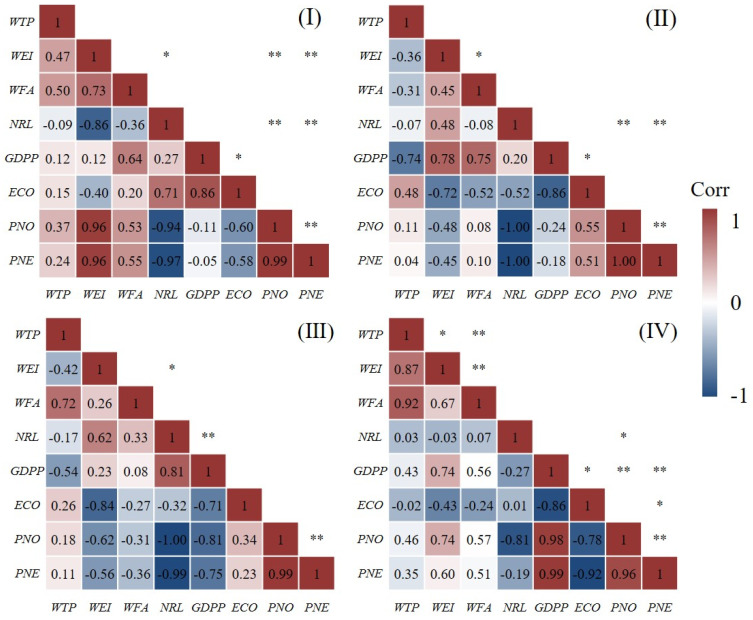
Correlation heat map of socioeconomic conditions in different farming types. “*” indicates significant correlation at the 0.05 level. “**” indicates significant correlation at the 0.01 level. (**I**) Hebei, Shandong, and Henan low-lying plains. (**II**) pre-mountain plain area in Yanshan and Taihang Mountains. (**III**) Huang-Huai plain area. (**IV**) Shandong hilly area.

**Figure 8:**
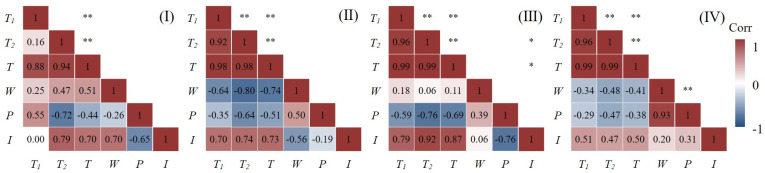
Correlation heat map of meteorological conditions in different farming types. “*” indicates significant correlation at the 0.05 level. “**” indicates significant correlation at the 0.01 level. (**I**) Hebei, Shandong, and Henan low-lying plains. (**II**) pre-mountain plain area in Yanshan and Taihang Mountains. (**III**) Huang-Huai plain area. (**IV**) Shandong hilly area.

Although the spatial pattern of wheat production is the result of the interaction of multiple factors, modeling all the selected variables will not only increase the complexity of the model but also lead to unstable analysis results and insignificant results of multicollinearity. Therefore, according to Equation (6) and with the help of SPSS software, the Spearman correlation test was performed on all variables, and variables with strong correlations were excluded. Considering the weak correlation between meteorological factors and socioeconomic conditions, the Spearman correlation test was carried out for both, separately for different farming areas, which made the test results more convincing (Figure 7 and Figure 8).

It can be seen from the figures that among the socioeconomic conditions, the pairs of variables with strong correlation are: *WFA*~*WTP*, *NRL~WEI*, *NRL~GDPP*, *PNE~PNO*, *GDPP~WEI*, *WTP~WEI, WEI~PNO*, *PNO~GDPP*, *PNE~GDPP*, *GDPP~WFA*, *ECO~PNE*, and *ECO~GDPP* (Figure 7). Among the meteorological conditions, the pairs of variables with strong correlation are: *T_1_*~*T*, *T_2_*~*T*, *T_1_*~*T_2_*, *W*~*P*, and *T_2_*~*I* (Figure 8). Based on previous research results and variable correlation results, six variables, *WEI*, *WFA*, *PNE*, *T*, *W* and *I*, were finally determined as the agricultural factors affecting winter wheat yield.

#### 3.2.2. Model Filtering

The HHH wheat production has entered a period of stable production since 2010, so 2010 and 2020 were appropriately selected as the comparison years. One task is to consider the availability and accuracy of the data, and the other is to facilitate spatial and temporal comparative analysis. Let the geographic center coordinate of the city i be $\left(u_{i},v_{i}\right)$; according to the variable screening results in Section 2.2.1 and Equations (7)–(10), the following SCA-GWR model is constructed:

(13) $$\begin{array}{ll} TGY_{i}= & \beta_{0}\left(u_{i},v_{i}\right)+\sum_{j=1}^{k}\beta_{1}\left(u_{i},v_{i}\right)x_{i1}\left(WEI\right)+\sum_{j=1}^{k}\beta_{2}\left(u_{i},v_{i}\right)x_{i2}\left(WFA\right)+\sum_{j=1}^{k}\beta_{3}\left(u_{i},v_{i}\right)x_{i3}\left(PNE\right)\\ & +\sum_{j=1}^{k}\beta_{4}\left(u_{i},v_{i}\right)x_{i4}\left(T\right)+\sum_{j=1}^{k}\beta_{5}\left(u_{i},v_{i}\right)x_{i5}\left(P\right)+\sum_{j=1}^{k}\beta_{6}\left(u_{i},v_{i}\right)x_{i6}\left(I\right)+\varepsilon_{i} \end{array}$$

Among the variables, $TGY_{i}$, $x_{i1}\left(WEI\right)$, $x_{i2}\left(WFA\right)$, $x_{i3}\left(PNE\right)$, $x_{i4}\left(T\right)$, $x_{i5}\left(P\right)$ and $x_{i6}\left(I\right)$ are the measured values of variables *TGY*, *WEI*, *WFA*, *PNE*, *T*, *P* and *I* at $\left(u_{i},v_{i}\right)$, and $\varepsilon_{i}$ is the error term.

The calculation of the regression coefficient is implemented in ArcGIS, the *AICc* method is selected for the model bandwidth calculation, and the Gaussian function is selected for the kernel function. The model parameters are shown in Table 2:

The SCA-GWR local model regression coefficient value varies from region to region, and it corresponds to a coefficient value for each prefecture-city unit variable in HHH. Table 3 is the descriptive statistics of the regression coefficients for variables in different cities at HHH in 2010 and 2020.

**Figure 9:**
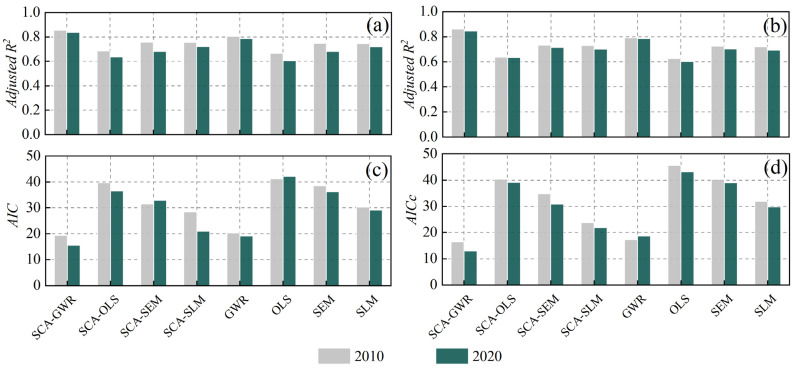
Model performance comparison. (**a**) *R^2^*; (**b**) adjusted *R^2^*; (**c**) *AICc*; (**d**) *AIC*.

To verify the necessity of using the SCA-GWR model and the spatial variability of the influencing factors of wheat production, according to the principle of the SCA-GWR model combined with the OLS, spatial residual model (SEM), and spatial lag model (SLM) related content [42], three global regression models, SCA-OLS, SCA-SEM, and SCA-SLM are constructed on the basis of correlation analysis. Considering that $R^{2}$ and adjusted $R^{2}$ can better characterize the fit of the regression equation, *AIC* and *AICc* can balance the complexity of the estimated model and the goodness of the fitted data. Therefore, the evaluation criteria $R^{2}$, adjusted $R^{2}$, *AIC*, *AICc*, and standard deviation are selected here for comparison analysis with the SCA-GWR model. The global regression analysis results (SCA-OLS, SCA-SEM, SCA-SLM) were obtained with the help of GeoDa software modeling (Table 4), and Table 3 is the output results of the local regression analysis. In addition, to verify the rationality of the relevant analysis, four traditional models, OLS, GWR, SLM, and SEM, are also included in the comparison range. Combined with Equations (11)–(12), the calculation results are shown in Figure 9.

Observing Table 2 and Table 4, it is found that the parameter estimates of the global regression analysis fluctuate greatly in different years, and the SCA-GWR model has a smaller standard deviation than the three global regression models, indicating that the parameter estimates of the SCA-GWR model are more stable. Although the global regression analysis in Table 4 eliminated variable multicollinearity, most parameter estimates failed the significance test.

Figure 9a,b confirm that the spatial regression is significantly better than the traditional nonspatial regression method. At the same time, the SCA-GWR model has the best fit, and the fitness indices are all above 0.8; the fit is relatively stable from 2010 to 2020. Since the input variables were screened for correlation analysis before the regression analysis, *AIC* and *AICc* of the SCA-GWR, SCA-OLS, SCA-SEM, and SCA-SLM models in Figure 9c,d were relatively low. Among them, the SCA-GWR model considers the spatial heterogeneity of variables based on optimizing the input variables, contains the fewest free parameters, and improves the simplicity of the model. Therefore, the *AIC* and *AICc* values of the SCA-GWR model are the smallest, both below 20.

The above analysis shows that the SCA-GWR local regression model is better than the traditional local regression model in explaining the influencing factors of the HHH wheat. Therefore, the following content will detect and analyze the influencing factors of the HHH wheat based on the results of the SCA-GWR model.

### 3.3. Detection of Influencing Factors for Wheat Production in the HHH Based on the SCA-GWR Model

**Figure 10:**
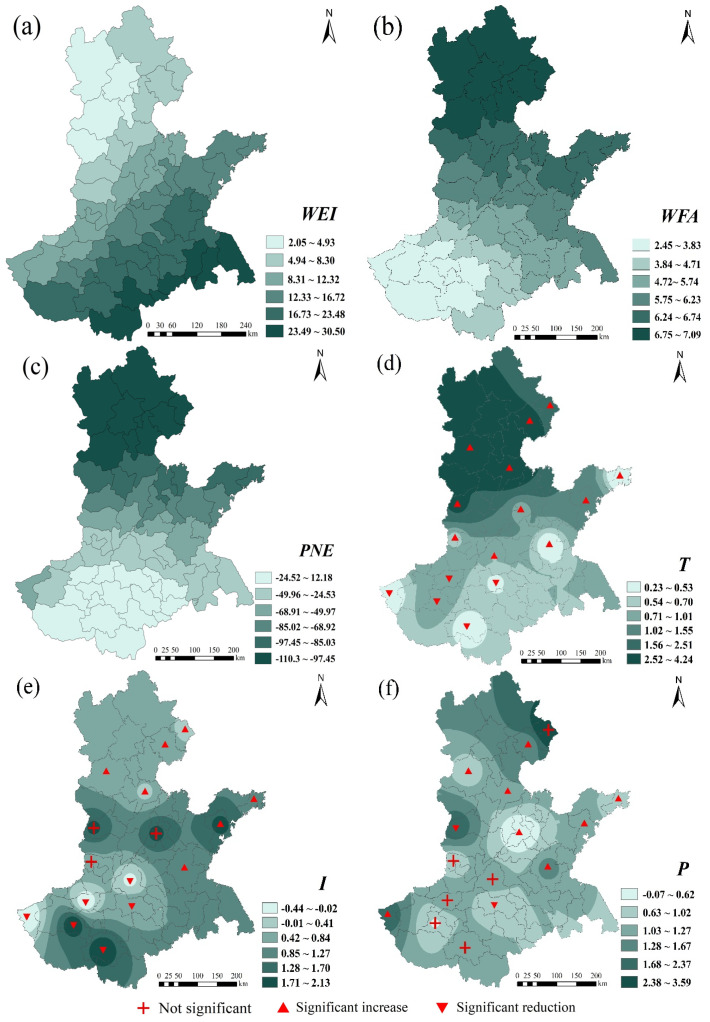
Spatial distribution of the regression coefficients for the SCA-GWR model in 2010: (**a**) *WEI*; (**b**) *WFA*; (**c**) *PNE*; (**d**) *T*; (**e**) *I*; (**f**): *P*.

**Figure 11:**
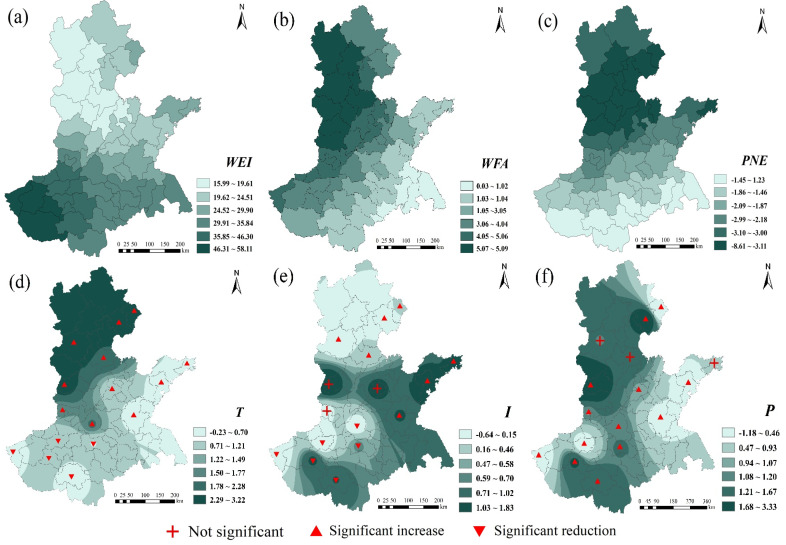
Spatial distribution of the regression coefficients for the SCA-GWR model in 2020: (**a**) *WEI*; (**b**) *WFA*; (**c**) *PNE*; (**d**) *T*; (**e**) *I*; (**f**): *P*.

To further explore the spatiotemporal influence mechanism of explanatory variables, we combined ArcGIS software to visualize the regression coefficients of explanatory variables in 2010 and 2020. The darker the color in the figure, the closer the corresponding variable is to wheat production in this area, and the red symbol represents the significant situation of the climatic tendency rate at each site (Figure 10 and Figure 11). In general, the spatial distribution of the regression coefficients of the 6 variables follows a certain law and is not random. The distribution patterns mainly include spatial clustering and gradient directionality. The specific analysis follows.

In 2010, the variable coefficients all passed the 1% significance test, that is, all regional local coefficients could explain the evolution of wheat production (Figure 10). The spatial distribution of the coefficients *WEI*, *WFA*, and *PNE* presents a spatial gradient pattern that gradually decreases from a certain direction. The effect of *WEI* on wheat decreased from south to north. *WFA* and *PNE* had a greater positive impact and inhibitory effect on wheat in northern HHH, respectively. Chu et al. used the geographic detector method to reach similar conclusions [43]. The spatial distribution of the regression coefficients of *T*, *I*, and *P* showed a certain clustering. The variables *T* and *P* both had a strong promoting effect on wheat production in Hebei Province, while the positive effect of the variable *I* was scattered mainly in the central and southern part of the HHH plain.

In 2020, the maximum *WEI* regression coefficient appeared in the mountainous and hilly areas of southwestern Henan province, and the minimum appeared in Zhangjiakou City, Baoding City, Shijiazhuang City, and other places in northwestern Hebei province (Figure 11). *WEI* and *WFA* in the HHH region were positively correlated with wheat production. *PNE* showed the distribution characteristics of “high in the middle and low in the north and south”. Meteorological factors *T*, *I*, and *P* have different effects on wheat production in different regions, and the direction of action is also different. The regression coefficients also have temporal and spatial differences and spatial agglomeration. This result verifies the research results of Wang et al. [44].

Combined with Equations (4)–(5), the spatial correlation test of the influencing factors of wheat production in HHH was further conducted (Table 5). In 2010 and 2020, most of the influencing factors of wheat yield in the municipal area had a significant positive correlation. Influenced by the topography of the HHH region and other natural geographic conditions, only *P* is not significantly correlated in space. The Moran’s I test results echo the distribution of the regression coefficients in Figure 10 and Figure 11.

In terms of socioeconomic conditions, the overall impact of *WEI* and *WFA* on wheat production in the HHH in 2020 increased compared with 2010. The regions with high *WFA* happened to have higher positive regression coefficients, which further indicated that the application of chemical fertilizers was very important for wheat cultivation. Within a certain range, increasing the number of chemical fertilizers can improve wheat yield [45]. During the study period, *PNE* was negatively correlated with wheat production in most of the central and northern regions of the HHH area. As an industrial province, Hebei province has a high *PNE*, and the industrialization and urbanization of rural villages and towns have hindered wheat production to a certain extent.

In terms of meteorological conditions, the average regression coefficient of *T* (1.35) is the highest (Table 3). Unlike 2010, *T* in 2020 has the opposite effect in the northern part of the HHH south (Figure 11). Winter wheat is a cool-loving crop. Combined with the climatic tendencies, there is a certain inhibitory effect on the growth of wheat in areas with high *T.* The low temperature in northern HHH provides the best conditions for winter wheat to overwinter. The distribution of the regression coefficients of *I* in 2010 was relatively uniform, but the regression coefficients in 2020 showed a spatial agglomeration trend, and the distribution state again verified the Moran’s I test results in Table 5. With time, the influence of *P* on wheat production increased, and gradually increased in northern HHH, mainly due to the drought trend in northern China in the past 10 years [46].

## 4. Discussion

Scientific and reasonable grain production patterns and correct regulation and control of production input are effective ways to ensure food security. Wheat is an important part of grain production. Exploring the spatial differentiation patterns of wheat production and the influencing factors can provide the decision-making basis for the adjustment of production structure and the allocation of factor resources. In this study, spatial analysis and SCA-GWR local modeling were used to quantify the spatial layouts and influencing factors of wheat. The main finding is that the contribution of different wheat producing areas to yield varies greatly, and the effects of different influencing factors on wheat vary from place to place.

In terms of the distribution of temporal and spatial differentiation patterns of wheat production, the study found that regional differences and inter-regional differences were consistently low in different farming areas (Figure 4). With the progress of science and technology, the popularization of agricultural mechanization has gradually expanded, and agricultural infrastructure in different farming areas has been constantly improved, so that the level of wheat farming in different regions has been synchronous improvement. Zhang et al. also found that the variation law of regional and interval differences was synchronous in time [47]. Due to regional differences and uneven resource allocation, wheat production in different regions presents a certain spatial agglomeration trend. In the study year, wheat production hotspots were concentrated in the southern part of the HHH and radiated to several surrounding municipalities, maintaining a “parallel” wheat production pattern (Figure 5). Hengshui city in the north of HHH maintained a high level of wheat production in 2013 and 2016, but the radiation effect on the surrounding area was not reflected. Zhang et al., by constructing a spatial weight matrix, found that wheat production in some areas of HHH has obvious spatial clustering characteristics, which is consistent with the results of this study [48]. Figure 6 depicts the overall spatial layout of wheat production. The center of gravity of wheat production in 2010 and 2020 is located in Henan province, and similar conclusions were reached by Li et al. [49]. Ji et al. also considered Henan province an important national grain production base, and the stability of wheat production in Henan province is of great significance to guarantee grain production in the central plains [50].

In terms of the detection of factors affecting wheat production, through the comparative analysis of regression coefficients in 2010 and 2020, it is found that among meteorological factors, *T* has the greatest impact on wheat production (Figure 10 and Figure 11). Light and temperature are the basis for photosynthesis and seed formation in winter wheat, and Jing et al. similarly identified *T* as the main force affecting crop variability and as being closely related to wheat growth and development [51]. The overall effect of *P* is small, mainly because the effect of precipitation is masked by irrigation conditions in most areas, which corroborates the findings of Luan et al. [52]. *P* has a greater impact on wheat production in the northern and southwestern parts of HHH, which are mountainous and hilly and not conducive to wheat cultivation, resulting in *P* being an important pathway for wheat to extract water. Cheng et al., have similar findings [53]. The regression coefficients were negative in regions where *I* was lower, indicating that lower *I* had a negative effect on wheat production, which is consistent with the findings of Xiao et al. [54]. Zhao et al. similarly concluded that *I* is positively correlated with wheat yield [55]. Among the socioeconomic conditions, *WEI* had the greatest impact on wheat, and *WEI* also compensated for the negative effect of the *P* deficiency. Wu et al. found that *WEI* will no longer be the limiting factor for yield if water supply is sufficient during the growing season [56]. The maximum value of the *PNE* coefficient occurs within the southwest, which has a high proportion of people employed in agriculture. The dense population and the large share of arable land in Henan province led to a strong correlation between *PNE* and grain production. *WFA* had a strong contribution to wheat production in both 2010 and 2020 in northern and northwestern HHH; however, Chen et al. found that *WFA* had the greatest effect on wheat production in central HHH through field experiments in 2014 [57]. In the present study, there was a gradual southward shift in the impact of fertilizer application on wheat production during the study period, a result that corroborates Chen et al. and reflects the dynamic changes in the impact of *WFA* on wheat production.

Wheat production is a complex process of multiple factors acting in response to time and place. Sustainable agriculture requires continuous institutional innovation. It is necessary to formulate different wheat production management policies and take different specific technical measures in accordance with the principles of adapting measures to local conditions and featuring prominent features. The research has the following policy implications:

(1) Zones I, II, and III were were the main contributors to the overall differences in HHH wheat. For wheat production, attention should be paid to the control of these regional differences, and measures should be taken according to local conditions, while strengthening the management of water and fertilizer to control and ultimately prevent agricultural endogenous pollution. The spatial agglomeration of wheat production is relatively strong (Figure 6). It is necessary to give full play to the learning and imitation abilities of farmers in neighboring regions, improve the technical efficiency of wheat production, and give full play to the planting advantages of different regions.
(2) Since *PNE* has an inhibitory effect on wheat in most areas (Figure 11), attention should be paid to the fluctuation in wheat planting areas caused by the transfer of agricultural labor in wheat production so as to effectively protect farmers’ income. According to the different effects of *T*, *I*, and *P* in the same area (Table 3), when optimizing the layout of wheat production, the spatial interaction of factors such as economic development and factor input should be fully utilized according to natural climatic conditions.
(3) Compared with 2010, wheat still relies heavily on *WEI* in 2020, while the demand for *WFA* is gradually weakening (Figure 10 and Figure 11). The rational use of water and fertilizer is the key factor in improving the utilization rate of water and fertilizer, which is related to the sustainable development of agriculture. Relevant management departments need to increase investment in agricultural infrastructure and high-standard farmland construction and promote the efficient and sustainable use of water and fertilizer resources. The scope of influence for *PNE* is gradually expanding. Against the background of continuous improvement in the nonagricultural labor force, the shortage of labor supply caused by the transfer of rural labor can be effectively dealt through the acceleration of agricultural mechanization and intelligent development.
(4) Over time, *T* has an inhibitory effect in some areas. With the gradual warming of the climate, it can delay the sowing time of wheat and slow down the growth and development rate before winter. In response to the problem of insufficient *I*, scientific and technological departments can vigorously develop radiation breeding technology based on environmental factors and wheat varieties to ensure the smooth progress of wheat photosynthesis. The previous analysis shows that *P* is only a part of the water supply for wheat, and irrigation is still needed to ensure the smooth growth of wheat. It is necessary to grasp the best irrigation period and amount of irrigation for wheat, improve the water use efficiency of wheat, and achieve sustainable development of high-yield, high-efficiency wheat.

To sum up, the formation process of wheat from seed germination to maturity to final yield is complex as the result of the combined effects of natural conditions and social and economic conditions. However, this paper assumes that technological progress and farmers’ behaviors are close to synchronization in various regions, which can be ignored. In addition, the study did not take into account natural conditions such as soil texture. We will comprehensively consider whether these factors can be ignored in follow-up research.

## 5. Conclusions

From the perspective of time, the overall level of wheat production tends to be the same, and the Hebei, Shandong, and Henan low-lying plains are the largest contributors to wheat production in HHH. From a spatial point of view, the center of gravity of wheat production is concentrated within Henan province and shows a high agglomeration. From the perspective of driving factors, *WEI* plays an important role in wheat production in different regions. The meteorological factors *T*, *I*, and *P* are also the basic necessities for wheat production.

In this paper, the Theil index, ESDA, and other methods were used to explain the spatial-temporal distribution characteristics of wheat production. The SCA-GWR local model was constructed on the basis of screening out six main factors, and its regression coefficients were expressed spatially differentially. Compared with the SCA-OLS, SCA-SEM, and SCA-SLM global models, the SCA-GER local model introduces a spatial matrix $\left(u_{i},v_{i}\right)$ that overcomes the nonstationarity of the spatial data and obtains regionalized regression coefficients. Compared with the traditional global models, OLS, SEM, and SLM, SCA-GER effectively avoids multicollinearity, improves model simplicity, and achieves the best fit. Through the comparison of the models, the superiority of the SCA-GWR model in explaining the spatial variation characteristics and laws of factors is verified, and new approaches are provided for explaining the spatial-temporal action mechanism of factors.

## Figures and Tables

**Table 1:** Variable descriptive statistics.

Year	Variable	Unit	Average	Maximum	Minimum	Upper Quartile	Lower Quartile	Median
2010	*TWY*	10^4^ ton	155.20	1098.46	107.04	221.68	66.52	131.85
	*WTP*	10^4^ kw	33,561.61	143,419.54	1987.59	44,264.45	15,090.82	27,522.41
	*WEI*	10^7^ m^2^	15,672.03	45,851.22	694.89	22,451.03	6730.75	12,639.72
	*WFA*	10^4^ ton	1528.18	4207.60	70.23	2202.16	713.55	1204.01
	*NRL*	10^4^	70.68	319.98	1.08	113.87	9.90	27.47
	*GDPP*	yuan	40,072.60	155,892.37	3648.24	49,575.96	17,799.53	33,679.70
	*ECO*	/	1.50	34.86	0.25	10.35	0.30	2.33
	*PNO*	%	84.14	160.40	84.30	95.30	96.15	120.30
	*PNE*	%	81.37	119.90	50.80	101.75	60.65	61.03
2020	*TWY*	10^4^ ton	190.98	1289.12	202.00	255.77	70.24	159.10
	*WTP*	10^4^ kw	33,881.96	121,197.22	1259.43	47,352.48	15,726.14	32,028.60
	*WEI*	10^7^ m^2^	18,812.90	54,031.37	1052.95	26,056.74	8197.97	15,171.26
	*WFA*	10^4^ ton	1628.59	3676.66	80.19	2502.11	763.71	1343.38
	*NRL*	10^4^	90.35	880.81	1.41	115.97	10.10	61.30
	*GDPP*	yuan	46,550.22	19,1173.06	894.77	66,501.80	4234.95	43,065.25
	*ECO*	/	1.18	29.60	0.14	0.30	0.24	0.27
	*PNO*	%	81.38	102.76	20.38	93.42	83.23	90.57
	*PNE*	%	77.20	126.64	53.19	89.39	64.16	70.67

Note: *TWY*: total wheat yield; *WTP*: weighted total power of agricultural machinery; *WEI*: weighted effective irrigated area; *WFA*: weighted fertilizer application scalar; *NRL*: number of rural labor force; *GDPP*: GDP per capita; *ECO*: Engel coefficient; *PNO*: proportion of nonagricultural industry output value; *PNE*: proportion of non-agricultural industry employment.

**Table 2:** SCA-GWR model parameter estimation and test results.

Model Parameters	2010	2020
Bandwidth	41.665	10.867
Residual Squares	50.44	66.12
Effective Number	16.297	17.021
Sigma	0.573	0.624
Degree of freedom	291.113	282.998
Residual Moran’s I	0.217 *	0.113 *

Note: “*” indicates that it passed the 1% significance test.

**Table 3:** SCA-GWR model regression coefficient descriptive statistics.

	Factor	Average	Maximum	Minimum	Upper Quartile	Lower Quartile	Median
2010	*WEI*	14.38	30.50	2.05	18.73	8.69	14.10
	*WFA*	4.17	7.09	2.45	6.65	3.85	5.25
	*PNE*	−68.14	12.18	−110.35	−39.64	−97.81	−85.32
	*T*	2.01	3.21	−0.24	2.35	0.62	1.48
	*P*	0.41	0.83	−1.78	0.18	−1.12	−0.47
	*I*	0.39	2.23	−2.48	2.05	−1.30	−0.125
2020	*WEI*	30.01	58.11	16.00	34.97	21.58	29.04
	*WFA*	0.06	5.09	0.03	0.07	0.05	0.06
	*PNE*	8.09	19.04	−3.11	3.12	−3.42	2.00
	*T*	1.35	3.22	−0.23	2.33	0.39	0.86
	*P*	0.48	1.83	−0.64	1.10	−0.24	0.48
	*I*	0.97	3.33	−1.18	1.55	−0.03	1.10

**Table 4:** Global regression analysis in 2010 and 2020.

SCA-OLS								
	2010				2020			
Variable	Coefficient	Standard Deviation	t/z Value	*p*-Value	Coefficient	Standard Deviation	t/z Value	*p*-Value
*intercept*	−8.476	5.976	−1.419	0.162	−4.659	9.946	−0.468	0.641
*WEI*	2.976	21.319	0.139	0.889	71.940	43.701	1.646	0.006
*WFA*	0.063	0.017	3.577	0.000	0.074	0.044	1.670	0.001
*PNE*	48.810	52.445	1.898	0.043	50.910	55.460	0.034	0.072
*T*	3.001	23.363	4.832	0.000	20.070	60.888	0.770	0.444
*P*	1.677	17.187	0.938	0.352	7.281	73.314	1.009	0.317
*I*	4.556	66.953	0.966	0.038	8.802	41.648	0.160	0.873
2010—F Statistics: 31.675, *p*-value: 0.023; 2020—F Statistics: 23.876, *p*-value: 0.876.								
**SCA-SEM**								
*intercept*	−8.587	5.336	−1.610	0.107	−7.291	7.266	−1.004	0.314
*WEI*	3.570	19.363	0.184	0.853	69.755	34.236	2.037	0.041
*WFA*	0.063	0.016	3.956	0.000	0.084	0.033	2.517	0.011
*PNE*	19.87	46.566	2.134	0.032	−22.193	42.458	−0.544	0.586
*T*	9.578	59.767	5.530	0.000	23.564	61.471	1.694	0.000
*P*	4.274	72.241	1.080	0.279	9.351	56.112	1.811	0.070
*I*	3.366	29.339	1.100	0.271	2.230	48.950	0.483	0.028
2010—Lambda: −0.045, Lagrange multiplier test: 10.987; 2020—Lambda: −0.675, Lagrange multiplier test: 19.013.								
**SCA-SLM**								
*intercept*	−7.356	5.606	−1.313	0.188	−5.129	8.950	−0.572	0.567
*WEI*	1.582	19.248	0.082	0.934	76.857	39.676	1.937	0.052
*WFA*	0.064	0.016	4.017	0.000	0.072	10.039	1.811	0.070
*PNE*	17.73	99.227	2.038	0.001	−52.761	18.150	−0.040	0.007
*T*	80.404	91.783	3.895	0.000	4.280	29.138	1.021	0.307
*P*	35.078	77.415	0.940	0.346	4.113	68.385	1.237	0.215
*I*	3.063	38.180	0.887	0.374	2.215	86.861	0.170	0.864
2010—Rho: 0.062, Lagrange multiplier test: 21.987; 2020—Rho: 0.591, Lagrange multiplier test: 30.013.								

**Table 5:** Global Moran’s I test of influencing factors for wheat production in the HHH from 2010 to 2020.

	2010				2020			
Factor	*Moran’s I*	Z Value	*p*-Value	Spatial Correlation	*Moran’s I*	Z Value	*p*-Value	Spatial Correlation
*WEI*	0.222	9.001	0.000	+	0.232	9.897	0.000	+
*WFA*	0.155	6.145	0.003	+	0.183	7.563	0.001	+
*PNE*	0.214	8.675	0.000	+	0.201	8.564	0.000	+
*T*	0.109	4.768	0.007	+	0.229	9.023	0.000	+
*P*	0.008	0.758	0.167	/	0.013	1.003	0.134	/
*I*	0.164	6.453	0.001	+	0.198	7.980	0.001	+

Note: “+” indicates a significant positive correlation, “/” indicates no significant correlation.

## Data Availability

Some or all data that support the finding of this study are available from the corresponding author upon reasonable request.
